# Chemical Analysis, Qualitative and Quantitative

**Published:** 1849-07

**Authors:** 


*Chemical Analysis, Qualitative and Quantitative.* By HENRY M. NOAD, Lecturer on Chemistry at St. George's Hospital, &c. *With Numerous Additions,* by CAMPBELL MORFIT, Practical and Analytical Chemist, &c. With illustrations, pp. 572. Philadelphia, LINDSAY & BLAKISTON, 1849.

The want of a work like the above, has been long felt, and few men were more eminently qualified to prepare such a one than the author of the present volume, and it certainly constitutes a most valuable acquisition to the literature of chemistry. It was prepared, as we are informed, by the American editor, as one of a series of chemical treatises for the "London Library of Useful Knowledge," and will be found highly valuable, not only to the analytical chemist, but to all engaged in the study of chemistry.

The work is divided into two parts: the first treats of qualitative analysis, and the second of quantitative. The introductory remarks in the English edition, upon chemical manipulation, have been omitted by the American editor, whilst he has introduced in its stead a chapter on equivalent proportions, chemical notation, and the general principles of chemical nomenclature.—*Balt. Ed.*

